# Levels of Trust in Information Sources as a Predictor of Protective Health Behaviors During COVID-19 Pandemic: A UAE Cross-Sectional Study

**DOI:** 10.3389/fpsyg.2021.633550

**Published:** 2021-07-21

**Authors:** Maria J. Figueiras, Jihane Ghorayeb, Mariana V. C. Coutinho, João Marôco, Justin Thomas

**Affiliations:** ^1^Department of Psychology, College of Natural Health and Sciences, Zayed University, Abu Dhabi, United Arab Emirates; ^2^Department of Psychology, College of Natural Health and Sciences, Zayed University, Dubai, United Arab Emirates; ^3^William James Center for Research, ISPA–Instituto Universitário, Lisbon, Portugal

**Keywords:** trust, protective health behaviors, sources of information, COVID-19, health, UAE

## Abstract

Health information sources and the level of trust in a particular source may influence the subsequent adoption of advocated health behaviors. Information source preference and levels of trust are also likely to be influenced by sociodemographic (culture, age, gender) variables. Understanding these source-trust-behavior relationships across various national and cultural contexts is integral to improved health messaging. The present study identified the sources most frequently consulted to obtain information about COVID-19 during the pandemic's early stages in the United Arab Emirates (UAE). The study quantified levels of trust across an array of information sources, factoring in sociodemographic variables. Finally, the study explored the relationship between sociodemographic variables, levels of trust in information sources, and the adoption of COVID-19 related protective behaviors. Participants (*n* = 1585) were recruited during the first 2 weeks of April 2020 via announcements in the UAE media and through email networks. All participants completed a web-based survey presented in English or Arabic, as preferred. The most frequently consulted information sources were websites (health information websites), social media, government communications, and family and friends. The sources rated most trustworthy were: personal physicians, health care professionals, and government communications. There were differences in the use of sources and levels of trust according to age, gender, and education. The levels of trust in sources of information were associated with the adoption of protective behaviors, significantly so for citizens of the UAE. These findings may help inform the improvement of pandemic–related health messaging in multicultural contexts.

## Introduction

The outbreak of the novel coronavirus disease (COVID-19) started in Wuhan, China, in the latter months of 2019, quickly spreading to other countries. The pathological presentation of COVID19 shared similarities with of MERS (Middle Eastern respiratory syndrome) and SARS (Severe Acute Respiratory Syndrome), including potentially fatal respiratory problems (Xu et al., 2020). In addition to a shortage in ventilators, inadequate enforcement of preventive measures, an absence of initial coordination among infected regions, and the unique communicability of COVID19 (long incubation period and high transmission rate) all contributed to the 2020 global pandemic (Peeri et al., 2020). Health authorities were charged with disseminating accurate information on protective behaviors to the general population to limit the spread of the disease. Hence, public health agencies in many countries, including the United Arab Emirates (UAE), encouraged personal protective measures (i.e., wearing masks and hand hygiene) along with interpersonal (i.e., social distancing), and internationally focused actions (i.e., travel restrictions) (Khosravi, 2020). However, some of these measures are difficult to enforce, especially when compliance is difficult to monitor (i.e., handwashing or sanitizing in private). Furthermore, as fear of contagion and generally anxiety spreads, so too do misconceptions about the virus and its transmission. Such misinformation can impact compliance with health-seeking and protective behaviors (Geldsetzer, 2020). The spread of misinformation can generate confusion, hinder public trust, and influence health-related behaviors. Within such a context ensuring accurate information becomes a paramount public health challenge (Limaye et al., 2020).

How emerging health information is selected, processed and evaluated during a pandemic will be influenced by numerous, potentially inter-related, factors. Such variables are likely to include the individuals' daily life circumstances, past experiences, culture, psychological risk orientations, traditions regarding health practices, reasoning strategies, and levels of trust in information sources (e.g., government vs. peers) (Vaughan and Tinker, 2009; Llewellyn, 2020). Studies conducted during previous pandemics identified numerous psychosocial variables potentially influential to the link between health information and engagement in protective behaviors. One factor that stood out as an essential predictor of such behaviors was level of trust in the source of health information (Liao et al., 2010; Bults et al., 2011; Blair et al., 2017). Higher levels of trust were associated with an increase in the probability of adoption of preventive measures, and mediated the relationship between information exposure and health behaviors.

Understanding levels of trust and protective behaviors in different socio-cultural contexts–the UAE included–is important, as findings from one society might not be applicable in another. For instance, collectivist cultural values, family size/structure, and governance systems might all influence the choice of information source, levels of trust, and protective health behaviors. Although far from homogeneous, UAE society has been characterized as being rooted in relatively collectivist values (Hofstede, 2001). Such collectivism can be expressed as a strong sense of familial and tribal interdependence. Such extended kinship (Qabeela) bonds remain relatively strong in the UAE (Al-Khazi, 2008) weekly (Friday) gatherings of the extended family are an expected routine for many citizens (Bristol-Rhys, 2010). Such cherished traditions and social norms might make it harder to adapt to the social/physical distancing requirements. Collectivist values are also commonly associated with living in larger family groups. Larger groups of people living in the same residence might accelerate the spread of the disease. Past research has found that household occupancy levels (people per house) were among the most important variables in predicting regional influenza epidemic severity (House and Keeling, 2009). More populous households had a greater likelihood of being infected and experienced higher internal transmission rates. This is based on the idea that, a larger number of household members increases the risk that one of them might bring the infection into the home, and more people under one roof is likely to mean a greater number of contacts. As a potential protective factor, collectivism is also associated with valuing group harmony and fitting in Hofstede (2001). Therefore, once social distancing becomes the norm, there is increased social pressure to obey the rules. This phenomenon has been referred to as the stringent norms hypothesis (Heinrichs et al., 2006).

Another factor that might impact trust and health behaviors during a pandemic is the nature of the nation's leadership and administration (e.g., Monarchy, Democracy, Autocracy). The UAE's system of governance, for example, facilitated a reasonably rapid response to the crisis. The Supreme Council, the top policy-making body in the UAE, has both legislative and executive powers (Embassy of the United Arab Emirates, 2020). Such powers ensure that the Supreme Council can plan and ratify federal laws rapidly when required, as was the case during the current pandemic.

For the above socio-cultural and demographic reasons, and due to the lack of previous regional research on this topic, a focus on the UAE could help shape future national pandemic preparedness plans and perhaps also inform those of neighboring Arab Gulf states. Socio-cultural context is an important factor to consider when exploring the determinants of health protective behaviors.

### Health Protective Behavior

During a pandemic, health protective behaviors can be categorized as preventive, avoidant, and management orientated (Bish and Michie, 2010). Preventive behaviors involve handwashing, sanitation, and mask-wearing. Avoidant behaviors include social distancing, avoiding crowded settings, and complying with quarantine and curfew measures. Management behavior consists of seeking medical advice from health professionals.

Studies from previous infectious disease outbreaks have identified several demographic and psychological factors associated with an increase in the adoption of protective behaviors. For instance, older and more educated individuals and women reported higher rates of compliance with hygiene practices and protective behaviors, compared to their younger, less educated, male counterparts (Agüero et al., 2011; Tooher et al., 2013; Moran and Del Valle, 2016). With regard to psychological determinants, again, researchers found that levels of trust in sources of health information was particularly important, along with the perceived risk of infection (Blair et al., 2017) and the perceived severity of symptoms (Tang and Wong, 2003; Agüero et al., 2011; Cairns et al., 2013; Tooher et al., 2013; Moran and Del Valle, 2016). In a recent review on public perception of a pandemic, Khosravi argued that the public perception of the pandemic, and the severity of the disease, facilitated feelings of vulnerability, which predicted a higher likelihood of adopting preventive measures (Khosravi, 2020). However, the focus of the paper was restricted to levels of trust targeted to sources of information among residents of the UAE, as well as type and amount of information sought; psychological variables linked to the illness impact such as perceived severity and perceived risk of the illness were considered beyond the scope of the article.

### Trust in Information Sources

The importance of trust as a predictor of protective behavior during disease outbreaks has been well-documented (Smith, 2006; Cairns et al., 2013; Fischhoff et al., 2018). For example, during the Ebola outbreak in Africa, researchers found that trust in authorities was positively associated with adherence to social distancing guidelines and seeking medical care in clinics in Liberia (Morse et al., 2016) and with vaccination compliance in the Congo (Blair et al., 2017). Similarly, recent research during the COVID-19 outbreak has pointed to the importance of trust as an enhancer of compliance with protective measures; in Australia, trust in health care professionals and scientists was associated with greater engagement in protective behaviors (Faasse and Newby, 2020).

Balog-Way and McComas suggested that transparency and the government's alignment with scientific experts were important for building trust during a pandemic (Balog-Way and McComas, 2020). These authors added that transparency was beneficial when people understood the risks and uncertainties of the outbreak (Birchall, 2011). Similarly, Khosravi reported that trust in the government to convey uncensored information also contributed to increased protective and preventive behaviors (Khosravi, 2020).

### Demographics and Trust in Sources of Information

Demographic variables such as age, income, and gender may influence online health-seeking behaviors (Rowley et al., 2017). Moreover, there is evidence that young and highly educated individuals tend to use and trust web-based information sources more often than older individuals. This may be due to young college-educated individuals having greater online information literacy and being better able to judge credibility cues more effectively (Liao and Fu, 2012; Rowley et al., 2017). Research also suggested that older (60+) individuals place less trust in internet sources compared to their middle-aged counter-parts, ranking internet and television as their least trusted information sources, followed by newspapers, friends and relatives, while their most trusted sources were health experts (e.g., pharmacists) (Le et al., 2014). The debate on gender differences is ongoing, with some studies suggesting that women trust online sources more than men, whereas other studies reveal no gender differences (Rowley et al., 2017).

As for the influence of culture, a cross-cultural study conducted in metropolitan cities of the US, Hong Kong, and South Korea revealed that individuals from these populations had high levels of trust in social media with HongKongers holding the highest levels of trust (Lin et al., 2016). Moreover, in a similar study comparing the same three groups, Song et al. found a significantly higher degree of trust for experience-based health knowledge (found in social network sites and blogs) in HongKongers and Koreans vs. Americans (Song et al., 2016). Furthermore, HongKongers and Koreans searched for experience-based knowledge as a source of health information more often than Americans.

In summary, past research has demonstrated that age, gender, education, and cultural differences can impact health behaviors and are associated with varying levels of trust in different information sources. Given the importance of information in managing pandemics, understanding the health information consumption habits of the population becomes particularly during such outbreaks. Although the COVID-19 pandemic is still ongoing at the time of writing, it is essential to understand how the perceived trustworthiness of information sources might influence the adoption of personal and interpersonal protective measures among the general public. An enriched understanding of such dynamics across various national and socio-cultural contexts can help inform pandemic-related health-messaging strategies. As such, the present study had the following aims: (1) To identify the sources (e.g., government, social media, mass media, interpersonal sources) most frequently consulted to obtain information about COVID-19 in the UAE, and the level of trust in those sources. (2) To examine the relationship between levels of trust in various information sources and the adoption of COVID-19 related protective behaviors.

## Materials and Methods

### Participants and Procedures

Participants (*n* = 1585) were recruited in April 2020 via announcements in the UAE media and through the email networks of UAE's National Program for Happiness and Wellbeing [National Programme for Happiness and Wellbeing (NPHW), 2020]. Additionally, the NPHW disseminated a link via their social media accounts on Twitter and Instagram. Under the direction of the Minister of State for Happiness, the NPHW also has a network of “happiness officers” scattered across more than 60 federal institutions, from universities to the police force; these happiness officers were also charged with dissemination of the study link via email blasts across their respective organizations. Inclusion criteria were (1) for participants to be residents of the UAE and (2) aged 18 years and above. The survey was written in English and translated (using the back-translation technique) to Arabic. The sample was not representative of the whole UAE, but did reflect many of its constituents. The mean age for the sample was 31.94 (*SD* = 11.59). Females made up 83.6% of the sample, and the two most populated emirates/city-states represented, Abu Dhabi and Dubai, accounted for 43.2 and 24.5% of the sample, respectively. The majority (65%) of the sample were citizens of the UAE (Emiratis). Datasets are available upon request.

### Ethics and Survey

An online survey was disseminated in early April 2020. Ethical approval was given by Zayed University Institutional and Review Board (R201213) and Ministry of Health and Prevention Research Ethics Committee (MOHAP/DXB-REC/ MMM/No. 49/2020). The survey included an online consent form–where participants had to click and agree to proceed—socio-demographic questions, sources of information, and level of trust in obtaining information about COVID-19, as well as the adoption of protective behaviors to reduce the risk of infection All questions in the present study were adopted, with permission, from previous research focused on this topic, specifically Shevlin et al. (2020). The items on trust in information sources used in the present study, and in Shevlin et al., were based on a review of the previous relevant literature. The same measures were also used by Murphy et al. (2021).

### Sociodemographic Variables

The sociodemographic characteristics included age, gender, education, and citizenship. Age was measured as a continuous variable. For the group comparison analysis, some of the variables were recoded. Age was recoded into four groups (18–24) (25–34) (35–44) (+45). Education was recoded into two groups (1 from primary to high school and 2 college/university). Citizenship was recoded as (1) UAE citizen (2) Non-UAE citizen.

### Sources of Information and Level of Trust

Two questions assessed the amount of information obtained from several sources and the level of trust in those sources: How much information about COVID-19 have you obtained from each of these sources? [ranging on a four-point Likert scale None (1) A little (2) Some (3) A lot (4)], and How much do you trust the information from each of these sources? (using the same Likert scale). The sources of information included were newspapers, TV, radio, websites, social media, personal doctors, other health care professionals, Government sources, and family and friends. Higher scores indicate a higher amount of information obtained and higher levels of trust in that source.

### Protective Behavior for COVID-19

A composite score of protective behavior for COVID-19 was computed by adding the variables measuring behaviors such as wearing a mask, respiratory etiquette, disinfecting surfaces, washing hands, and using sanitizer. These five behaviors were selected as they are part of the WHO's recommendations for limiting the spread of COVID-19 (World Health Organization, 2021). Responses to the protective behavior items were as follows: 1 (no) 2 (occasionally) and 3 (regularly).

### Statistical Analysis

Descriptive and non-parametric analyses for ordinal data (Wilcoxon, Mann-Whitney, and Kruskal-Wallis tests) were used to probe the use of sources of information and trust tabulated by socioeconomic variables. The choice of non-parametric tests (i.e., Wilcoxon and Mann-Whitney) also addressed the limitation of the sample, unequal group size and unmet parametric assumptions (Field, 2018). For paired comparisons, Wilcoxon test was used. An ordinal regression analysis was carried out to probe the predictive value of the level of trust in sources of information and citizenship in predicting the adoption of protective behavior, after controlling for age, gender, and education (covariates). Considering that residents and citizens of the UAE come from different cultural backgrounds, which may affect the adoption of protective behaviors, citizenship was added as a predictor in the model. The dependent variable (adoption of protective behaviors) was ordinal with three response categories. The predictor variables, level of trust in different sources of information, were recoded into dummy variables (yes/no) and checked for multicollinearity. The proportional odds assumption was tested with the parallel lines test [χ^2^(117) = 131.64, *p* = 0.168]. All statistical analyses were performed with IBM SPSS Statistics, version 26 (Armonk. IBM Corp, 2017). Results were considered statistically significant for *p* ≤ 0.05. For each group comparisons tests *post hoc* power calculations were performed using Gpower 3.1 (Faul et al., 2007). For a medium effect size at *p* < 0.05, the achieved power was 99%.

## Results

**Figure 1:**
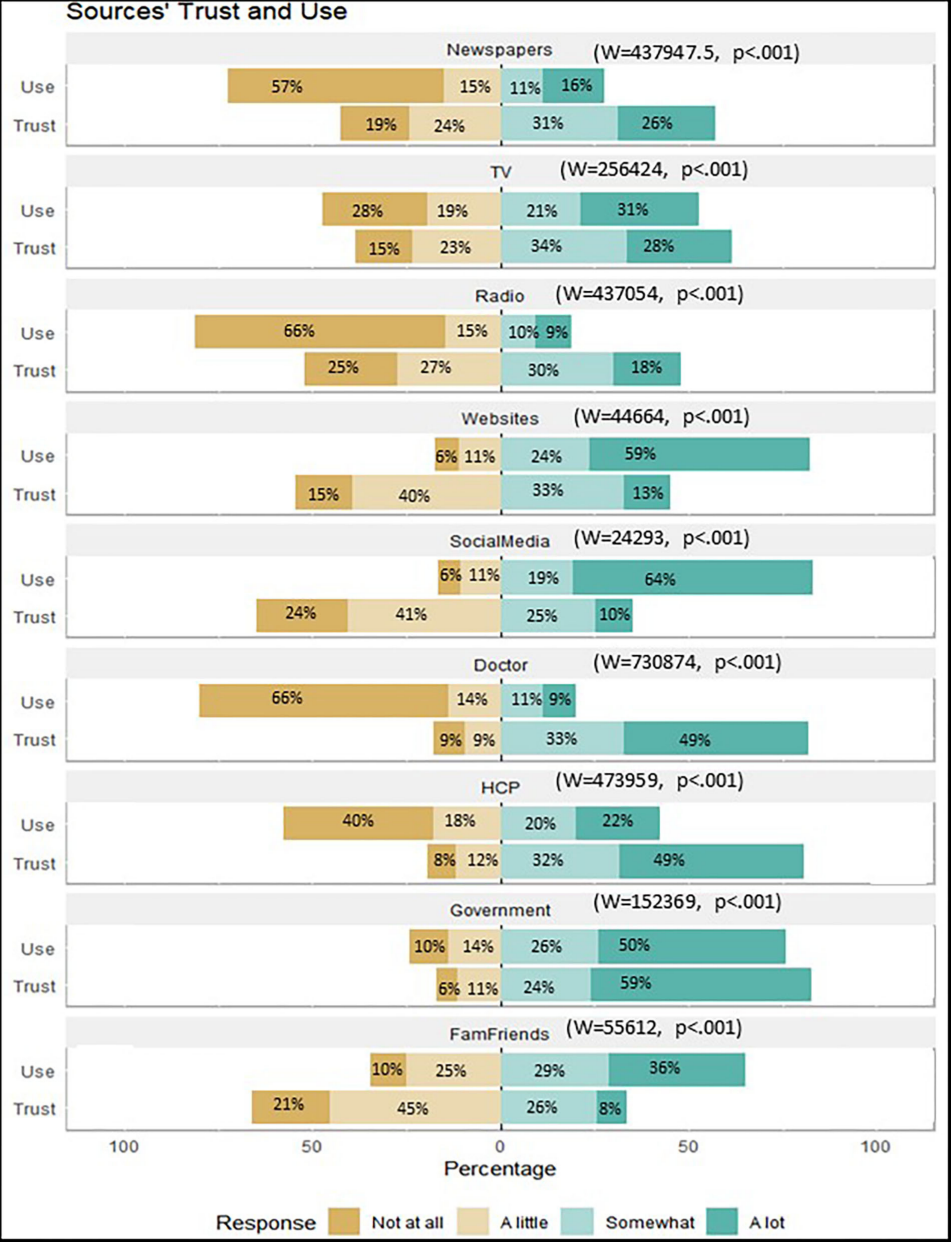
Amount of information obtained from different sources and level of trust.

To identify the sources of information mostly frequently sought, and corresponding levels of trust, a descriptive analysis was used. This was also broken down by demographic variables (Figure 1). The most frequently consulted sources of information were social media, websites, government sources, and family and friends. The sources considered more trustworthy were government sources, personal doctors, other health care professionals, and TV. Differences between the amount of information obtained from the sources and the level of trust were tested using a Wilcoxon test for paired samples. There was a significant difference between the amount of information obtained from all the different sources and the level of trust in those sources (Figure 1). Participants mentioned frequent use of websites (*W* = 44664, *p* < 0.001, social media (*W* = 24293, *p* < 0.001) and family and friends (*W* = 55612, *p* < 0.001), but with low levels of trust. Conversely, participants reported seeking less information from newspapers (*W* = 437947.5, *p* < 0.001), TV (*W* = 256424, *p* < 0.001), radio (*W* = 437054, *p* < 0.001), personal doctors (*W* = 730874, *p* < 0.001), health care professionals (*W* = 473959, *p* < 0.001), and the government (*W* = 152369, *p* < 0.001), however, in these sources they expressed a higher level of trust.

### Differences According to Sociodemographic Variables

#### Gender

There were statistically significant differences according to the U Mann-Whitney test between males and females on the mean ranks of information obtained from newspapers (*U* = 146759.0; *p* < 0.001), radio (*U* = 158906.0; *p* = 0.043), social media (*U* = 148937.5; *p* < 0.000), health care professionals (*U* = 149416.0; *p* = 0.006), government sources (*U* = 151038.5; *p* = 0.004), and family and friends (*U* = 131796.0; *p* < 0.001), as well as in the level of trust in TV (*U* = 151404.0; *p* = 0.007) and social media (*U* = 148899.5; *p* = 0.002). Females obtained more information than males, except for newspapers and radio sources, and reported higher levels of trust in TV and social media than males (Tables 1, 2).

**Table 1:** Differences in sources of information among sociodemographic variables.

**Variables Mean ranks (Median)**		**N**	**Newspapers**	**TV**	**Radio**	**Websites**	**Social media**	**Personal doctors**	**Other health care professionals**	**Government**	**Family and friends**
Gender^a^	Females	1,325	760.50 (1)	783.50 (3)	770.71 (1)	786.36 (4)	805.08 (4)	778.49 (1)	793.33 (2)	800.14 (4)	811.62 (3)
	Males	260	845.47 (2)	771.28 (3)	790.92 (1)	781.14 (4)	706.78 (4)	766.35 (1)	712.16 (2)	716.70 (3)	644.85 (3)
	U		146759.00	164551.00	158906.00	168644.00	148937.50	162269.00	149416.00	151038.50	131796.0
	Signif.		0.000^***^	0.068	0.043^*^	0.84	0.000^***^	0.639	0.006^**^	0.004^**^	0.000^***^
Age group^b^	18–24 yrs. old	608	661.59 (1)	737.66 (2)	695.27 (1)	777.50 (4)	909.54 (4)	690.48 (1)	737.64 (2)	860.88 (4)	941.67 (4)
	25–34 yrs. old	345	730.89 (1)	731.59 (2)	751.27 (1)	793.85 (4)	784.56 (4)	813.88 (1)	819.04 (2)	779.83 (3.5)	722.17 (3)
	35–44 yrs. old	389	875.82 (2)	826.43 (3)	844.48 (1)	806.50 (4)	727.42 (4)	846.90 (1)	802.88 (2)	738.78 (3)	713.15 (3)
	+45 yrs. old	243	964.04 (2)	891.20 (3)	894.31 (1)	760.02 (4)	591.86 (3)	828.19 (1)	793.81 (2)	686.47 (3)	590.35 (3)
	H (3)		128.04	29.79	65.01	2.42	129.54	52.15	9.97	37.62	145.48
	Signif.		0.000^***^	0.000^***^	0.000^***^	0.491	0.000^***^	0.000^***^	0.019^*^	0.000^***^	0.000^***^
Education^a^	Primary to high school	452	672.54 (1)	746.24 (2)	711.41(1)	758.51(4)	905.50 (4)	699.26 (1)	700.72 (2)	832.32 (4)	939.39 (4)
	University degree	1,133	814.86 (1)	795.37 (3)	798.88 (1)	796.18(4)	742.63 (4)	807.06 (1)	811.17 (2)	768.35 (3)	722.93 (3)
	U		198666.5	231629.0	216000.0	238301.0	200926.5	210654.0	211297.5	230662.000	181125.0
	Signif.		0.000^***^	0.045^*^	0.000^***^	0.093	0.000^***^	0.000^***^	0.000^***^	0.006^**^	0.000^***^
Citizenship^a^	UAE	1,023	683.61 (1)	767.11 (3)	729.21 (1)	787.08 (4)	874.93 (4)	740.47 (1)	766.12 (2)	831.85 (4)	864.62 (3)
	Non-UAE	562	937.57 (2)	807.61 (3)	854.74 (1)	782.63 (4)	632.95 (3)	841.22 (1)	804.84 (2)	703.87 (3)	638.66 (3)
	U		184996.0	264954.0	230049.5	280744.5	197370.5	240746.0	265616.5	236652.0	200250.0
	Signif.		0.000^***^	0.078	0.000^***^	0.833	0.000^***^	0.000^***^	0.088	0.000^***^	0.000^***^

a *Mann-Whitney test*.

b *Kruskal-Wallis test*.

* *p < 0.05*,

** *p < 0.01*,

*** *p < 0.001*.

**Table 2:** Differences in level of trust in sources of information among sociodemographic variables.

**Variables Mean ranks (median)**		**N**	**Newspapers**	**TV**	**Radio**	**Websites**	**Social media**	**Personal doctors**	**Other health care professionals**	**Government**	**Family and friends**
Gender^a^	Females	1,325	785.79 (1)	798.10 (3)	781.23 (1)	780.66 (4)	798.66 (4)	776.94 (1)	786.23 (2)	787.63 (4)	774.03 (3)
	Males	260	753.42 (2)	718.12 (3)	767.68 (1)	785.75 (4)	706.63 (4)	783.45 (1)	772.58 (2)	765.30 (3)	822.71 (3)
	U		159482.50	151404.00	163631.00	166601.00	148899.50	163843.50	164885.00	162512.50	156875.50
	*p*		0.277	0.007^**^	0.648	0.862	0.002^**^	0.818	0.632	0.415	0.093
Age group^b^	18–24 yrs. old	608	859.18 (3)	843.48 (3)	787.04 (2)	746.54 (2)	825.36 (2)	800.10 (4)	819.60 (4)	887.42 (4)	729.00 (2)
	25–34 yrs. old	345	692.27 (2)	722.94 (3)	734.99 (2)	758.66 (2)	783.79 (2)	773.69 (3)	775.01 (4)	730.58 (4)	778.68 (2)
	35–44 yrs. old	389	756.84 (3)	750.11 (3)	804.42 (3)	832.94 (3)	765.10 (2)	756.85 (3)	760.27 (4)	718.68 (4)	820.10 (2)
	+45 yrs. old	243	743.53 (3)	781.99 (3)	780.42 (2)	818.74 (2)	706.46 (2)	762.51 (3)	745.13 (4)	704.99 (3)	858.81 (2)
	H (3)		36.51	20.25	5.06	12.39	14.07	3.10	7.87	65.94	20.42
	*p*		0.000^***^	0.000^***^	0.171	0.006^**^	0.003^**^	0.376	0.049^*^	0.000^***^	0.000^***^
Education^a^	Primary to high school	452	856.68 (3)	857.26 (3)	790.61 (2)	747.87 (2)	826.83 (2)	823.36 (4)	833.07 (4)	874.29 (4)	743.65 (2)
	University degree	1,133	750.10 (3)	756.30 (3)	774.38 (2)	794.86 (2)	766.19 (2)	759.99 (3)	764.48 (3)	747.96 (4)	797.27 (2)
	U		214188.5	218202.0	241607.5	233264.5	230729.5	225926.0	228099.5	209959.0	231688.0
	*p*		0.000^***^	0.000^***^	0.505	0.050^*^	0.012^*^	0.006^**^	0.003^**^	0.000^***^	0.024^*^
Citizenship^a^	UAE	1,023	798.00 (3)	819.60 (3)	779.10 (2)	763.68 (2)	820.53 (2)	791.67 (4)	796.06 (4)	833.52 (4)	742.77 (2)
	Non-UAE	562	748.90 (3)	722.14 (3)	778.82 (2)	813.84 (2)	716.79 (2)	753.37 (3)	762.06 (3)	694.20 (3)	853.04 (2)
	U		261544.0	246828.5	277505.5	261496.0	244163.5	263831.0	268861.0	231265.5	240445.0
	*p*		0.033^*^	0.000^***^	0.990	0.026^*^	0.000^***^	0.079	0.123	0.000^***^	0.000^***^

a *Mann-whitey test*.

b *Kruskal-Wallis test*.

* *p < 0.05*

** *p < 0.01*

*** *p < 0.001*.

#### Age

There were significant differences according to the Kruskal-Wallis test by age group (original variable recoded in four groups) on the amount of information obtained from newspapers [*H*(3) = 128.04; *p* < 0.001], TV [*H*(3) = 29.79; *p* < 0.001], Radio [*H*(3) = 65.01; *p* < 0.000], social media [*H*(3) = 129.54; *p* < 0.001], personal doctors [*H*(3) = 52.15; *p* < 0.001], other health care professionals [*H*(3) = 9.97; *p* = 0.019] government [*H*(3) = 37.62; *p* < 0.001] and family and friends [*H*(3) = 145.48; *p* = 0.000]. Younger groups of participants obtained fewer amounts of information from newspapers, TV, radio, and personal doctors than the older groups. Younger groups used significantly more social media, government sources, and family and friends' sources compared to older groups (Table 1). Concerning levels of trust, younger groups reported higher levels of trust in newspapers [*H*(3) = 36.51; *p* < 0.001], TV [*H*(3) = 20.25; *p* < 0.001], websites [*H*(3) = 12.39; *p* = 0.006], social media [*H*(3) = 14.07; *p* = 0.003], health care professionals [*H*(3) = 7.87; *p* = 0.049], government [*H*(3) = 65.94; *p* = 0.000], and less trust in family and friends [*H*(3) = 20.42; *p* = 0.000], in comparison with older groups (Table 2).

#### Education

There were also differences in the levels of trust and amount of information obtained from different sources based on participants' level of education. For analysis purposes, the original variable was recoded into two levels (1–primary to high school) and (2–college/university). The Mann-Whitney test showed significant differences in all sources of information, except for websites. Participants with higher levels of education (university diploma and postgraduates) reported obtaining more information from newspapers (*U* = 198666.5; *p* < 0.001), TV (*U* = 231629.0; *p* = 0.045), radio (*U* = 230049.5, *p* < 0.001), and personal doctors (*U* = 240746.0; *p* < 0.001) and fewer amounts of information from social media (*U* = 197370.5; *p* < 0.001), government sources (*U* = 236652.0; *p* < 0.001), and family and friends (*U* = 200250.0; *p* < 0.001) (Table 1). Concerning trust, participants with higher levels of education (university diploma and postgraduates) reported lower levels of trust in newspapers (*U* = 214188.5; *p* < 0.001), TV (*U* = 218202.0; *p* < 0.001), social media (*U* = 230729.5; *p* = 0.012), personal doctors (*U* = 225926.0; *p* = 0.006), other health care professionals (*U* = 228099.5; *p* = 0.003), government sources (*U* = 209959.0; *p* < 0.001), and higher levels of trust in websites (*U* = 233264.5, *p* = 0.050) and family and friends (*U* = 231688.0; *p* < 0.001) (Table 2).

#### Citizenship

For citizenship, according to the Mann-Whitney test, significant differences were found in the amount of information obtained from newspapers, radio, personal doctors, social media, government, and family and friends. Local citizens obtained more information from social media (*U* = 244163.5; *p* < 0.001), government (*U* = 236652.0; *p* < 0.001), and family and friends (*U* = 200250.0; *p* < 0.001) (Table 1) than their non-citizen (expatriate) counterparts. For levels of trust, significant differences were found in newspapers, TV, websites, social media, government, and family and friends. Local citizens reported higher levels of trust in newspapers (*U* = 261544.0; *p* = 0.033), TV (*U* = 246828.5; *p* < 0.001), social media (*U* = 244163.5; *p* < 0.001) and government (*U* = 231265.5; *p* < 0.001), and lower levels of trust in websites (*U* = 261496.0; *p* = 0.026) and family and friends (*U* = 240445.0; *p* < 0.001), compared to non-UAE citizens (Table 2).

#### Predictors of Protective Behaviors

To determine whether levels of trust in sources of information were predictors of the adoption of protective behaviors for COVID-19, an ordinal regression model was used. The level of trust in sources of information, and the citizenship of residents (UAE vs. Non-UAE) were used as predictors of the probability of adopting protective behavior for COVID-19, after controlling for age, gender, and education (two groups). Results showed that an increase in the level of education (from high school to university level) was associated with an increase in the odds of adopting protective behavior for COVID-19 [OR = 1.56 (95% CI, 1.292–1.880), Wald χ^2^(1) = 21.604, *p* < 0.000]. Participants with higher education were 56% more likely to adopt preventive behaviors. No significant effect of gender as a covariate was found. Trust in information from social media and government sources increased the probability of adopting protective behaviors to prevent infection [OR = 1.23 (95%CI, 1.020–1.488), Wald χ^2^(1) = 4.702, *p* < 0.03]; [OR = 1.38 (95%CI, 1.113–1.702), Wald χ^2^(1) = 8.733, *p* = 0.003], respectively. Participants who trusted social media and government sources were 23 and 38%, (respectively) more likely to adopt protective behaviors to reduce the risk of infection from COVID-19 than those who did not trust these sources. Being a citizen of the UAE reduced the probability of adopting protective behavior for COVID-19 [OR = 0.81 (95%CI, 0.666–0.991), Wald χ^2^(1) = 4.180, *p* < 0.041]. UAE citizens were 19% less likely to adopt protective behaviors to reduce the risk of infection from Covid-19 (Table 3).

**Table 3:** Estimates, standard-errors, significance, odd ratio, and 95% confidence intervals for the ordinal regression model.

**Ordinal regression (logit)**		**Estimate**	**SE**	**Wald**	**df**	***p***	**95% Confidence interval**		**OR**	**OR 95% Confidence interval**	
							**Lower bound**	**Upper bound**		**Lower bound**	**Upper bound**
Covariates	Age	0.013	0.005	7.756	1	0.005^**^	0.004	0.023	1.01	1.003	1.022
	Gender	−0.071	0.111	0.411	1	0.521	−0.288	0.146	0.93	0.749	1.157
	Education	0.444	0.096	21.604	1	0.000^***^	0.257	0.632	1.56	1.292	1.880
Predictors level of trust	Newspapers	0.004	0.103	0.001	1	0.972	−0.199	0.206	1.00	0.819	1.229
	TV	0.044	0.107	0.165	1	0.685	−0.167	0.254	1.04	0.846	1.288
	Radio	0.068	0.103	0.435	1	0.510	−0.134	0.269	1.07	0.874	1.308
	Websites	0.131	0.088	2.192	1	0.139	−0.042	0.304	1.14	0.958	1.355
	Social media	0.209	0.096	4.702	1	0.030^*^	0.020	0.398	1.23	1.020	1.488
	Personal doctors	0.085	0.132	0.420	1	0.517	−0.173	0.344	1.09	0.841	1.410
	Health care professionals	−0.045	0.129	0.125	1	0.724	−0.298	0.207	0.96	0.742	1.229
	Government	0.320	0.108	8.733	1	0.003^**^	0.108	0.532	1.38	1.113	1.702
	Family and friends	−0.084	0.088	0.915	1	0.339	−0.255	0.088	0.92	0.774	1.091
	Citizenship	−0.207	0.101	4.180	1	0.041^*^	−0.406	−0.009	0.81	0.666	0.991

* *p < 0.05*

** *p < 0.01*

*** *p < 0.001*.

## Discussion

The present study had two main aims. The first was to identify the sources most frequently used for COVID-19 information in the UAE and assess the levels of trust in those information sources. The second aim was to examine the relationship between levels of trust in particular information sources and the adoption of protective behaviors. Significant differences were found across age groups, educational levels and between genders for the amount of information obtained from specific sources, and in the levels of trust placed in those sources. Furthermore, high levels of trust in social media and government communications, as well as participants' citizenship (UAE vs. Non-UAE citizens), positively predicted the adoption of protective behaviors after controlling for the effects of age, gender, and education.

Consistent with previous research, the present study found that the most consulted sources of information for COVID19 varied by sociodemographic variables. Women obtained more information from social media, health care professionals, government communications, family, and friends compared to men. Women also reported higher levels of trust in social media and TV than men. Concerning age, younger adults obtained less information from traditional media (i.e., newspapers, TV, radio) and more from social media, government communications, and family and friends compared to the older groups. Education level was also associated with the use of sources of information, as well as the level of trust in those sources. Specifically, participants with higher levels of education reported obtaining more information from mass media and health care professionals, and less from social media, government, family, and friends. Surprisingly, individuals with more education reported higher levels of trust in family and friends compared to government, doctors and healthcare professionals. Perhaps the role of culture and collectivistic values was influential here, with familial trust being an essential ingredient in fostering group harmony. The specific role of cultural values merits further investigation in the context of COVID-19 and the UAE.

Interestingly, the most frequently consulted sources of information were not necessarily perceived as the most trustworthy ones, which is in line with several recent studies that also reported paradoxical and counter-intuitive relationships between information source, trust and protective behavior (Le et al., 2014; Lin et al., 2016). One COVID-19 related study highlighted a “trust paradox,” in which a high level of public trust in the government, and concomitant low levels of perceived risk, resulted in low compliance with the government's risk management measures. This brought to light the challenges in explaining the discrepancy between trust and the use of information from different sources. It calls for further reflection on how psychological variables such as perceived risk, and perceived severity of the illness, influence public trust and compliance with protective behaviors (Wong and Jensen, 2020). The present study did not assess severity perceptions, and this is discussed further in the limitations section.

Trust in social media and government were significant predictors of the adoption of protective behaviors. This finding was consistent with a previous study showing that trust in formal sources of information (government/media) about influenza (H1N1) was associated with higher reported hand hygiene (Bults et al., 2011). This finding was also in line with previous studies during the Ebola outbreak in Africa, showing that trust in governmental authorities positively predicted the adherence to social distance guidelines, seeking medical care, and getting vaccinated (Liao et al., 2010; Agüero et al., 2011). In this regard the current findings are aligned with past literature showing that trust plays an essential role in fostering high levels of concordance with recommended health measures (Vaughan and Tinker, 2009; Khosravi, 2020). The present study also found that citizenship status was associated with adopting protective behaviors. UAE citizens were less likely to adopt protective behavior for COVID-19 than Non-UAE citizens. This is hard to explain, but perhaps, family structure, a strong sense of familial interdependence among citizens might have made it harder to adapt to the social/physical distancing requirements. Furthermore, cultural activities, such as gatherings and greeting behavior can be difficult to alter and individuals may not readily avoid them, reducing the adoption of protective behaviors (Bruns et al., 2020). Another possibility is that non-citizens have more to lose by falling foul of the authorities. The sanctions for non-compliance might pose a greater threat to the livelihood of individuals with employment-related residence visas. Possible interventions should address cultural beliefs and assumptions to ensure that communication of information about protective behaviors is culturally appropriate.

Trust in social media and its association with the adoption of protective behavior may be attributed to how resources such as Facebook, Twitter, Instagram, Google+, and other social tools have created innovative opportunities to transmit and exchange health-related knowledge (Murphy et al., 2021). According to the Cambridge English dictionary (Cambridge International Dictionary of English, 1995), social media also known as participative media, refers to web-based applications that enable users to create and share content and participate in social networking, typically by responding to each other's content. For example, people can easily share information from different sources through social media, including scientific findings and government information. These platforms enabled people to compare the messages given by various sources and draw their individual conclusions on them, which might in turn influence their level of trust in the information source. Future studies could investigate further the specific online sources consulted, especially given the proliferation of smartphones and a myriad social media channel.

With regard to the relationship between trust in government communications and the adoption of protective behaviors reported in the study, partnerships formed between UAE government and several health care providers may have played a role here. Furthermore, the nature of the nation's leadership and administration may impact positively on trust and health behaviors during a pandemic. Previous research suggested that when governmental entities collaborated with health care providers in providing information about the risks of a pandemic and the benefits of compliance with protective actions, they were more effective in controlling the spread of the disease (World Health Organization, 2021). Future research should address how an interdisciplinary trust model could provide guidance on how to translate trust into a protective behavior.

The present study has several important limitations. The cross-sectional nature did not allow us to investigate changes in behavior over time. An opportunistic, non-representative sample of the UAE population cannot be considered representative and there are several constituents notably absent e.g., manual laborers. Most of the participants in the study were females between 18 and 34 years of age. Another limitation is that we did not differentiate between websites and newspapers that could be accessed online, these categories are possibly conflated for some respondents. Furthermore, trust and credibility were considered as similar aspects of the same concept, even though the two terms are not always seen as interchangeable. Some authors consider these terms as interchangeable and synonymous while others believe they are distinct (Sbaffi and Rowley, 2017). Sbaffi et al. reviewed the different views on trust, and when reported credibility to be subjective to the individual and not reflective of the actual accuracy and veracity of a content. In addition, Corritore et al. (2003) discussed different levels of trust needed to be assessed to determine which information could be translated into action. Had this paper split the concept of trust into different levels or distinguished between credibility and trust maybe a clearer understanding of the paradox would have emerged as to why some participants trusted certain sources of information but sought others more often. Moreover, psychosocial variables such as risk perception and perceived severity of the illness were not included in the study. Risk and severity may influence the perception of vulnerability and how individuals trust sources of health information to adopt protective behavior; hence these variables ought to be included in future research within this population. Despite these limitations, the present study contributed to a deeper understanding of the role of sources of information and trust in predicting the adoption of protective behavior for COVID-19 in the UAE context.

In summary, the results of this study suggest that health messaging during a pandemic may benefit from using various communication channels, while simultaneously adapting message content based on the sociodemographic status of the individuals most likely to utilize and or trust a given source/channel. While further research is required, these findings have practical implications and could help improve and refine pandemic–related health communications in highly multicultural societies such as the UAE.

## Data Availability Statement

The raw data supporting the conclusions of this article will be made available by the authors upon request. Requests can be directed to the corresponding author.

## Ethics Statement

The study as approved by Zayed University Institutional and Review Board (R201213) and Ministry of Health and Prevention Research Ethics Committee (MOHAP/DXB-REC/MMM/No. 49/2020). The patients/participants provided their written informed consent to participate in this study.

## Author Contributions

MF contributed to the conceptualization of aims, goals and writing draft and revision, formal analysis, and methodology as well as interpretation. JG contributed to the project administration, writing of the literature draft review and editing, as well as formatting for submission. MC contributed to the writing of literature review, drafting, revision, and editing. JM contributed to the formal analysis and interpretation, drafting of the results section, figures, and revision. JT contributed to the conceptualization of the survey and dissemination, data acquisition, data curation, drafting, and revision. All authors contributed to the article and approved the submitted version.

## Conflict of Interest

The authors declare that the research was conducted in the absence of any commercial or financial relationships that could be construed as a potential conflict of interest.
